# Chemical Composition and Biological Activities of Essential Oils from the Leaves, Stems, and Roots of *Kadsura coccinea*

**DOI:** 10.3390/molecules26206259

**Published:** 2021-10-16

**Authors:** Tianming Zhao, Chao Ma, Guofei Zhu

**Affiliations:** College of Food and Pharmaceutical Engineering, Guizhou Institute of Technology, Guiyang 550003, China; machao@git.edu.cn (C.M.); zhuguofei@git.edu.cn (G.Z.)

**Keywords:** *Kadsura coccinea*, essential oil, chemical composition, antioxidant, antimicrobial, cholinesterase inhibitory

## Abstract

The chemical composition and biological activities of the essential oils from the leaves, stems, and roots of *Kadsura coccinea* (*K. coccinea*) were investigated. The essential oils were extracted by hydro distillation and analyzed by gas chromatography mass spectrometry (GC-MS) and gas chromatography with flame ionization detector (GC-FID). Antioxidant activities of the essential oils were examined with DPPH radical scavenging assay, ABTS cation radical scavenging assay, and ferric reducing antioxidant power assay. Antimicrobial activities were evaluated by determining minimum inhibitory concentrations (MIC) and minimum microbiocidal concentrations (MMC). Acetylcholinesterase and butyrylcholinesterase inhibitory activity of the essential oils were also tested. A total of 46, 44, and 47 components were identified in the leaf, stem, and root oils, representing 95.66%, 97.35%, and 92.72% of total composition, respectively. The major compounds of three essential oils were α-pinene (16.60–42.02%), β-pinene (10.03–18.82%), camphene (1.56–10.95%), borneol (0.50–7.71%), δ-cadinene (1.52–7.06%), and β-elemene (1.86–4.45%). The essential oils were found to have weak antioxidant activities and cholinesterase inhibition activities. The essential oils showed more inhibitory effects against *Staphylococcus aureus* (*S. aureus*) than those of other strains. The highest antimicrobial activity was observed in the root oil against *S. aureus*, with MIC of 0.78 mg/mL. Therefore, *K. coccinea* essential oils might be considered as a natural antibacterial agent against *S. aureus* with potential application in food and pharmaceutical industries.

## 1. Introduction

*Kadsura coccinea (K. coccinea)* (Lem.) A. C. Smith is an evergreen climbing shrub and belongs to the Schisandraceae family. It is mainly distributed in China and Vietnam. In China, it is widely spread in southern provinces, such as Jiangxi, Guizhou, Guangxi, Guangdong, Sichuan, and Yunnan. In folk medicine, the dried roots and stems of this plant are commonly known as “Heilaohu” in Chinese and has been used for the treatment of chronic gastritis, bruises, rheumatism, and dysmenorrhea [1]. Over the past two decades, extensive studies have been performed on the chemical compounds from roots and stems of *K. coccinea*. In particular, lignans [2,3,4,5,6] and triterpenoids [7,8,9,10,11,12] from this plant have been received considerable attention. In previous studies, the compounds of *K. coccinea* have been reported with various activities, including the inhibition of nitric oxide production [2,13], antioxidant [14,15], anti-HIV [16], anti-inflammatory [17], hepatoprotective [18], acetylcholinesterase and butyrylcholinesterase inhibitory activities [15,19,20], and other pharmacological effects.

Essential oils are rich in bioactive compounds and have gained popularity because of their various activities including antioxidant, antimicrobial, anti-inflammatory, and different enzyme inhibition activities. However, reports on the chemical characterization of essential oils of *K. coccinea* and their activities are limited. The chemical composition of the essential oil from the stem barks of *K. coccinea* collected in Vietnam was reported, with β-caryophyllene (23.6%), δ-cadinene (8.6%), caryophyllene oxide (7.8%), epi-α-bisabolol (7.5%), and α-copaene (6.6%) as the major compounds [21]. The essential oil of *K. coccinea* collected in Hunan province (China) was found to be toxic to the Bed Bug and its major compounds were β-caryophyllene (24.73%), caryophyllene oxide (5.91%), α-humulene (3.48%), and β-pinene (2.54%) [22]. Previous studies also reported the volatile components in different parts of *K. coccinea* [23,24] and evaluated the antioxidant activity of essential oils using DPPH radical scavenging assay [23]. In order to obtain a more comprehensive picture about antioxidant potential of *K. coccinea* essential oils, several methods with different mechanisms should be employed. To the best of our knowledge, no information on the antimicrobial and enzyme inhibitory activities of *K. coccinea* essential oils was available. *K. coccinea* showed acetylcholinesterase and butyrylcholinesterase inhibitory activities which could be attributed to some lignans and triterpenoids, it is interesting to find out whether its essential oils possess the same activities.

The chemical composition of essential oils could be influenced by many factors including environmental conditions, geographic variations, physiological variations, genetic factors, etc. [25]. The essential oils of the same plant from different geographic locations could be quite different in chemical composition. The essential oil chemical compositions of *K. coccinea* collected in Guiyang (Guizhou province, China) have not been reported and the results could contribute to better understanding the diversity of chemical composition of the essential oils from this plant.

In this study, essential oils from the leaves, stems, and roots of *K. coccinea* were prepared and analyzed using gas chromatography-mass spectrometry (GC-MS) and gas chromatography (GC) with flame ionization detector. Antioxidant activities were evaluated by DPPH radical scavenging assay, ABTS cation radical scavenging assay and ferric reducing antioxidant power assay. Antimicrobial activities against several microorganisms were evaluated by determining minimum inhibitory concentrations (MIC) and minimum microbiocidal concentrations (MMC). Acetylcholinesterase and butyrylcholinesterase inhibitory activity assays were also performed.

## 2. Results

### 2.1. Chemical Composition of K. coccinea Essential Oils

The essential oils from the leaves, stems, and roots of *K. coccinea* are light yellow, transparent liquids. The yields (*w*/*w*) of the different oil samples of *K. coccinea* ranged from 0.17% to 0.74%. The highest yield was observed for the leaf oil (0.74 ± 0.03%), followed by the root oil (0.44 ± 0.02%) and the stem oil (0.17 ± 0.005%).

The chemical composition of the oils isolated from the different parts (leaves, stems, and roots) was determined by GC and GC/MS. The chemical compositions of the essential oils were presented in Table 1. A total of 46 components were identified in the leaf oil representing 95.66% of total composition in which α-pinene (42.02%), β-pinene (18.82%), β-elemene (4.45%), myrcene (2.93%), β-caryophyllene (2.87%), and β-phellandrene (2.05%) were identified as major compounds. For the stem oil, 44 identified components accounted for 97.35% of total composition and the major compounds were α-pinene (41.79%), β-pinene (18.71%), camphene (4.43%), 3-carene (3.39%), δ-cadinene (3.06%), myrcene (2.89%), β-caryophyllene (2.71%), α-copaene (2.29%), β-elemene (2.04%), and β-phellandrene (1.99%). A total of 47 constituents were identified in the root oil accounting for 92.72% of the total composition. The dominant compounds were α-pinene (16.60%), camphene (10.95%), β-pinene (10.03%), borneol (7.71%), δ-cadinene (7.06%), β-caryophyllene (3.08%), α-copaene (3.03%), 3-carene (2.43%), eucalyptol (2.23%), and α-thujene (2.07%).

In the essential oils from the leaves, stems, and roots of *K. coccinea*, a total of 60 components were identified and 33 components existed in all three essential oils. Mono- and sesquiterpene hydrocarbons dominated in the leaf and stem oils. The percentages of monoterpene hydrocarbons were 72.84% for the leaf oil and 77.8% for the stem oil. The percentages of sesquiterpene hydrocarbons were 15.49% and 14.34%, respectively. The main fraction of the root oil was monoterpene hydrocarbons (48.38%), followed by sesquiterpene hydrocarbons (22.29%), oxygenated monoterpenes (11.79%), and oxygenated sesquiterpenes (9.67%).

### 2.2. Antioxidant Activity of K. coccinea Essential Oils

Antioxidant activity is a complex process usually occurring through several mechanisms and its evaluation is often carried out by more than one test method [26]. Antioxidant activities of the *K. coccinea* essential oils were determined using the DPPH radical scavenging assay, ABTS cation radical scavenging assay, and ferric reducing antioxidant power assay. BHT (butylated hydroxytoluene) was used as the positive control. The results are expressed as Trolox equivalents (TE) and could be seen in Table 2. FRAP assay gave higher Trolox equivalents values than DPPH and ABTS assays. For the essential oils from different parts of *K. coccinea*, the root oil showed the highest antioxidant activity, followed by the stem oil and leaf oil. In comparison with positive control, *K. coccinea* essential oils showed significantly lower radical scavenging capacities against DPPH and ABTS radicals and reducing ability (*p* < 0.05). These results indicated that *K. coccinea* essential oils displayed very weak antioxidant effects.

### 2.3. Antimicrobial Activity of K. coccinea Essential Oils

The results of antimicrobial activity assay of the essential oils from the leaves, stems, and roots of *K. coccinea* were shown in Table 3. It can be seen that the essential oils showed inhibitory activities against all the microorganisms tested. The MICs of the essential oils against *Staphylococcus aureus (S. aureus)*, *Staphylococcus lentus (S. lentus)*, *Pseudomonas aeruginosa (P. aeruginosa)*, *Escherichia coli (E. coli)*, and *Candida albicans (C. albicans)* ranged from 0.78 mg/mL to 6.25 mg/mL. The highest antimicrobial activity was observed in the root oil against *S. aureus*, with MIC of 0.78 mg/mL. However, the essential oils displayed much weaker inhibitory activities against *Bacillus atrophaeus (B. atrophaeus)*, with MICs of 50 mg/mL, 100 mg/mL, and 25 mg/mL, respectively. In comparison with the positive controls, the essential oils of *K. coccinea* displayed weak antimicrobial activity against the microbial strains tested.

### 2.4. Cholinesterase Inhibition Activity of K. coccinea Essential Oils

Alzheimer’s disease (AD) is known as a neurodegenerative disorder and inhibition of acetyl/butyrylcholinesterase is the most common strategy for treatment of AD [27]. Cholinesterase inhibitory activities of *K. coccinea* essential oils were presented in Table 4. At the final concentration of 2 μg/mL, essential oils of *K. coccinea* did not exert inhibitory activity against acetylcholinesterase (AChE) and butyrylcholinesterase (BChE). At the final concentration of 20 μg/mL, the leaf and root oils did not show inhibitory activity against butyrylcholinesterase (BChE). The positive controls (tacrine and iso-OMPA), however, had the inhibition percentages of 55.39% and 48.54% at very low concentration of 0.33 μg/mL and 0.86μg /mL, respectively. The results indicated that *K. coccinea* essential oils showed weak cholinesterase inhibition activity. IC_50_ values against acetylcholinesterase (AChE) and butyrylcholinesterase (BChE) were not reported in this study, as some of them were difficult to obtain.

## 3. Discussion

Differences in the qualitative and quantitative composition of the essential oils obtained from the leaves, stems, and roots of *K. coccinea* have been observed. However, the chemical compositions of the leaf and stem oils are more similar than that of the root oil. No difference (*p* > 0.05) was found between the relative percentages of five major components (α-pinene, β-pinene, myrcene, β-caryophyllene, and β-phellandrene) in the leaf and stem oils. The compounds with significant difference (*p* < 0.05) in the leaf and stem oils were camphene (1.56% and 4.43%), β-elemene (4.45% and 2.04%), α-copaene (0.89% and 2.29%), δ-cadinene (1.52% and 3.06%), etc. 3-carene was not identified in the leaf oil, but it was found with an amount of 3.39% in the stem oil. The chemical profile of the root oil was mainly characterized by higher percentages of oxygenated monoterpenes and oxygenated sesquiterpenes. Borneol had a relative percentage of 7.71% in the root oil, but it accounted for less than 1% in the leaf and stem oils.

Only a few papers dealing with the chemical composition of *K. coccinea* essential oil are available in literature. The components in this study was obviously different from the essential oil reported by Dai et al. [21], who found that the major compounds were β-caryophylene, δ-cadinene, caryophyllene oxide, and α-copaene. The components were also different from those reported by Rehman et al. [22], who determined that the major compounds were β-caryophyllene, caryophyllene oxide, α-humulene, and β-pinene. However, the essential oils reported by Yang et al. [23] showed several same major compounds as our research, such as pinene, δ-cadinene, and β-elemene. The results indicated that the essential oil components of *K. coccinea* from different geographic regions were different.

In comparison with different species in the genus *Kadsura*, some similarities and differences were noticed. The major compounds (δ-cadinene, camphene, borneol, and cubenol) in the essential oil of *Kadsura longipedunculata* [28] have been identified in the present work. The three abundant compounds in the essential oils of *Kadsura oblongifolia* [29] were cis-cadina-1,4-diene, α-coapene, and β-caryophyllene, whose percentages were much lower in our research. α-eudesmol from *Kadsura heteroclita* [30] was the most abundant volatile which was not identified in this study. In another publication on *Kadsura heteroclita* [31], δ-cadinene, calarene, and δ-4-carene were found to be the major compounds, which were different from our results.

A number of reports are available in the literature on the antioxidant capacity of the essential oils and their individual constituents until now. In general, phenolic compounds such as thymol, eugenol, and carvacrol showed good antioxidant activity due to their high reactivity with radicals [32]. In this study, no phenolic compounds were identified, which may partially justify the weak antioxidant activities of the *K. coccinea* essential oils. The antioxidant activities of the oils could be described by the presence of the main bioactive components of the oils. For example, it has been reported that terpenes such as α-terpinene, γ-terpinene, sabinene, terpinolene, and limonene showed antioxidant activities [33,34]. Some oxygenated monoterpenes and sesquiterpenes—such as terpinen-4-ol, α-terpineol, borneol, and spathulenol—also exhibited antioxidant activities [33,35,36]. The root oil showed higher antioxidant activity than the stem and leaf oils, which could be explained by its higher contents of oxygenated monoterpenes (11.79%) and oxygenated sesquiterpenes (9.67%). It is noteworthy that α-pinene was reported to be capable of preventing lipid peroxidation [37]. The *K. coccinea* essential oils were characterized by the high content of α-pinene. Further studies are required to confirm the potential of the oils in lipid peroxidation prevention.

To our knowledge, this is the first study reporting on the antimicrobial activity of the essential oils from *K. coccinea*. Of the microorganisms tested, *S. aureus* was the most sensitive strain to the essential oils of *K. coccinea*, confirmed by the lower MICs. *S. aureus* is one of the most common pathogenic bacteria in nature, which has caused numerous diseases, becoming a serious threat to human health. Meanwhile, the abuse of antibiotics aggravated the harmfulness of *S. aureus*. The essential oils of *K. coccinea* exhibited more inhibitory effects against *S. aureus* than those of other microbial strains, indicating their potential use as nature-based antimicrobial agent. Particularly, the root oil showed lower MIC against *S. aureus* than the leaf and stem oils. This could be explained by the difference in the chemical composition of the leaf, stem, and root oils. The root oil had higher percentages of oxygenated monoterpenes and oxygenated sesquiterpenes, which could contribute more to the antimicrobial activity. Borneol, known as a natural antibiotic which is present in many medicinal plants [38], had a relative percentage of 7.71%, 0.50%, and 0.63% in the root, leaf, and stem oils, respectively. For the antimicrobial mechanism of the essential oils, *S. aureus* was used as a model strain in the previous study [39] and the disruption of cell structures, such as the cytomembrane, has been confirmed by cytomembrane permeability, protein and K^+^ leakage, and electron microscopy. Similarly, the essential oils of *K. coccinea* could exert antimicrobial activity by damaging cell structure.

It is noteworthy that *B. atrophaeus* showed the resistance to the essential oils of *K. coccinea*. *B. atrophaeus* belongs to the genus *Bacillus* which is known for its high resistance to heat, chemical reagents and radiation, etc. *B. atrophaeus* could even produce some bioactive metabolites, such as antifungal compounds reported in the previous study [40], which means that *B. atrophaeus* could co-exist with these bioactive compounds. Due to its high resistance, this bacterial species is often used as a biological indicator of sterilization or disinfection [41]. Medicinal plants are an excellent source of antimicrobial or microbiocidal substances. In the test of disinfection or microbiocidal agents against microorganisms, *B. atrophaeus* is also used as a representative for spore-forming bacteria, some of which were pathogenic. In the present study, *B. atrophaeus* was used for the evaluation of the antimicrobial or microbiocidal action of *K. coccinea* essential oils against spore-forming bacteria. The results indicated very weak antibacterial activity against this species.

Differences in cholinesterase inhibition activity were observed in the essential oils from different parts of *K. coccinea*. The leaf and stem oils seemed to have stronger acetylcholinesterase inhibition activities than the root oil at the concentration of 20 μg/mL. The difference could be explained by the presence of the main components in these essential oils. The previous study reported that α-pinene and β-pinene exhibited AChE inhibitory activity [42]. The leaf and stem oils had much higher percentages of α-pinene and β-pinene than the root oil, which could be the reason for the difference.

## 4. Materials and Methods

### 4.1. Plant Materials

Leaves, stems, and roots of *K. coccinea* were collected in August 2020 from the Mountain Jiulong near Guiyang (Latitude 26°39′37″ N, longitude 106°32′23″ E, altitude 1400 m). Plant material was dried at room temperature in the shade for about three weeks before extraction of essential oils. Identification was made by associate Prof. Yazhou Zhang (School of Pharmacy, Guizhou University of Traditional Chinese Medicine, Guiyang, China) according to the Flora of China and voucher specimens were deposited at the Laboratory of Pharmaceutical Engineering, Guizhou Institute of Technology.

### 4.2. Chemicals

*n*-alkanes C7-C30, DPPH (2,2-diphenyl-1-picrylhydrazyl), and Amphotericin B were purchased from Sigma-Aldrich (St. Louis, MO, USA). Trolox (6-hydroxy-2,5,7,8-tetramethylchroman-2-Carboxylic Acid), BHT (tert-butyl hydroxytoluene), and TPTZ(2,4,6-Tripyridyl-s-triazine) were purchased from Shanghai Yuanye Biotechnology Co., Ltd. (Shanghai, China). ABTS (2,2′-Azino-bis (3-Ethylbenzothiazoline-6-Sulfonic Acid) was from Beijing Solarbio Science & Technology Co., Ltd. (Beijing, China). Gentamycin sulfate was purchased from Shanghai Aladdin Biochemical Technology Co., Ltd. (Shanghai, China). *n*-hexane and methanol were from Tianjing Fuyu Fine Chemicals Co., Ltd. (Tianjing, China). DMSO was from Tianjing Kemiou Chemical Reagent Co., Ltd. (Tianjing, China). Potassium persulfate was from United Initiators (Shanghai, China). All other analytical grade chemicals were from Sinopharm Chemical Reagent Co., Ltd. (Shanghai, China).

### 4.3. Extraction of Essential Oils

The dry leaves (150 g), stems (600 g), and roots (150 g) of *K. coccinea* were ground into small pieces, then subjected to hydro distillation in Clevenger-type apparatus for 5 h using 3 L, 6 L, and 3 L of deionized water, respectively. Each extraction was conducted in three replications. The essential oil yield was calculated on a dry-weight basis (*w*/*w*). The oil samples were stored in amber-colored glass bottles at −20 °C for further analysis.

### 4.4. GC-MS and GC-FID Analysis

The *K. coccinea* essential oils were diluted 1:50 *v*/*v* in *n*-hexane and analyzed on the GC-MS and GC-FID systems. The identification of essential oil components was carried out using a GC-MS (TQ8040 NX, Shimadzu, Yokohama, Japan) equipped with an InertCap WAX capillary column (60 m in length, 0.25 mm in inner diameter, and 0.25 μm in film thickness) and an AOC-6000 auto-sampler (Shimadzu, Yokohama, Japan). Column temperature was programmed initially at 50 °C, then increased to 110 °C with a rate of 2 °C/min, and then increased to 240 °C with a rate of 3 °C/min. Helium was used as the carrier gas with a flow rate of 1 mL/min, and the volume of injection was 1μL in split mode (1:10). MS parameters were as follows: the mass range was 10–550, the ion source temperature was 230 °C and the interface temperature was 250 °C. The retention indices (RI) of the components were determined with a homologous series of *n*-alkanes (C7–C30) under the same operating conditions. Finally, the essential oil components were identified by comparison of their RIs and experimental mass spectra with those published in the literature [43] and NIST Mass Spectral libraries.

The quantification of essential oil components was carried out on a GC-Trace 1310 (Thermo Scientific, Waltham, MA, USA) with FID detector. The GC column and temperature program were the same as described for the GC-MS system. The injector temperature was 250 °C and the detector temperature was 250 °C. The nitrogen was used as the carrier gas and the volume of injection was 1 μL in split mode (1:10). The relative percentage (%) of each component in the essential oils was obtained by peak area normalization without using correction factors. The GC analysis was performed in triplicate for each essential oil of *K. coccinea.*

### 4.5. Antioxidant Activities

#### 4.5.1. DPPH Radical Scavenging Assay

Scavenging activity of essential oils against stable DPPH radical was determined following the method previously described by Brand-Williams et al. [44] with some modifications. Essential oil solutions of 20 mg/mL were prepared using methanol as solvent. For further DPPH scavenging assessment, the more effective essential oils were diluted. The solution of DPPH in methanol (6 × 10^−5^ mol/L) was prepared daily, before measurements. 6 mL of this solution was mixed with 150 μL of essential oil solution and the mixed solution was put in the darkness for 30 min. The absorption was then read at 515 nm on the UV–vis spectrophotometer (UH5300, HITACHI, Tokyo, Japan). Radical Scavenging Activity (RSA) was calculated by the formula:

$$\mathrm{Inhibition}\;\mathrm{Percentage}\;\left(\%\right)=\frac{A_{B}-A_{A}}{A_{B}}\times 100\%$$

in which: *A_B_* = absorbance of DPPH solution (t = 30 min); *A_A_* = absorbance of tested essential oil solution (t = 30 min).

The methanol solutions of Trolox with known concentrations ranging from 100 to 750 μmol/L were used for calibration. The antioxidant activity of essential oil was expressed in μmol Trolox equivalents (TE)/g of essential oil. BHT was used as the positive control.

#### 4.5.2. ABTS Cation Radical Scavenging Assay

ABTS cation radical scavenging activity was determined according to the method described by Xu et al. [45] with some modifications. Briefly, the ABTS cation radical was generated by mixing 5 mL of ABTS (7 mmol/L) with 88 μL of potassium persulfate (140 mmoL/L), followed by reaction in the dark overnight at room temperature. The ABTS cation radical solution was diluted with methanol to an absorbance of 0.700 ± 0.050 at 734 nm. 50 μL of essential oil solution in methanol was mixed with 4 mL of diluted ABTS solution. The mixture was allowed to stand for 6 min at room temperature, and the absorbance was immediately recorded at 734 nm. Radical scavenging activity of the essential oils was calculated. The antioxidant activity of essential oil was expressed in μmol Trolox equivalents (TE)/g of essential oil and BHT was used as the positive control.

#### 4.5.3. Ferric Reducing Antioxidant Power Assay

The ability of essential oil to reduce ferric ion to ferrous ion, ferric reducing/antioxidant power (FRAP) assay was performed according to a slightly modified method performed by Benzie and Szeto [46]. Working FRAP reagent was prepared by mixing 50 mL acetate buffer (300 mmol/L, pH 3.6), 5 mL TPTZ solution (10 mmol/L), and 5 mL FeCl_3_•6H_2_O solution (20 mmol/L). The FRAP reagent was warmed to 37 °C before use. 150 μL of essential oil solution was mixed with 450 μL of methanol, and then 4.5 mL of FRAP reagent. The mixture was allowed to stand at 37 °C and the absorbance was recorded at 593 nm in 30 min. The results were expressed in μmol Trolox equivalents (TE)/g of essential oil and BHT was used as the positive control.

### 4.6. Antimicrobial Activities

The essential oils of *K. coccinea* were tested against several microorganisms. The bacteria assayed included: (i) three Gram-positive bacteria, namely: *S. aureus* ATCC 6538, *S. lentus* BNCC 185251, and *B. atrophaeus* ATCC 9372; and (ii) two Gram-negative bacteria, namely: *P. aeruginosa* ATCC 15442 and *E. coli* BNCC 185254. The yeast used was *C. albicans* ATCC 10231. Bacterial strains were cultured in Luria-Bertani (LB) broth agar medium and *C. albicans* was grown in yeast maltose medium.

The minimum inhibitory concentrations (MIC) and minimum microbiocidal concentrations (MMC) were detected following the method previously described by Tian et al. [47] with some modifications. When each strain was cultivated to the logarithmic growth stage, the culture concentration was diluted to 10^6^ CFU/mL. Essential oils of *K. coccinea* were dissolved in 20% dimethyl sulfoxide (DMSO)-80% distilled water for an initial concentration of 200 mg/mL, 120 μL of which was firstly added to a 96-well plate. Then serial dilutions (100~0.39 mg/mL) were obtained by 2-fold dilution method described by Baltzar [48]. 60 μL of diluted microbial strain suspension was added. The plates were incubated at 37 °C for 16–24 h for the bacteria and at 28 °C for 48 h for the yeast. 20% DMSO-80% distilled water without essential oils was used as negative control. 

After reading the MIC results, 60 μL of the suspension from the wells in which there was no visible growth was pipetted and evenly spread on agar plates. The plates were incubated at 37 °C for 16–24 h for the bacteria and at 28 °C for 48 h for the yeast. The MMC was determined from MIC results. The minimum microbiocidal concentration (MMC) was defined as the lowest recorded essential oil concentration of the MIC wells in which strains failed to grow. The gentamycin sulfate and amphotericin B were used as standard antibacterial and antifungal drugs for the determination of their MIC and MMC.

### 4.7. Cholinesterase Inhibition Activities

Enzyme inhibitory properties of the essential oils of *K. coccinea* connected to neurodegenerative disease were investigated against acetylcholinesterase (AChE) and butyrylcholinesterase (BuChE), using the methods previously described by Sun and Yang [49,50]. Briefly, 10 μL of essential oil solution was mixed with 40 μL of phosphate buffer (pH 7.4), 20 μL of DTNB (5,5’-Dithiobis-2-nitrobenzoic acid, 2.5 mmol/L or 5 mmol/L), and 10 μL of AChE (or BuChE) solution in a 96-well microplate. The mixture was incubated at 37 °C for 10 min. The reaction was then initiated with the addition of 20 μL of acetylthiocholine iodide (10 mmol/L) or butyrylthiocholine iodide (25 mmol/L). The reaction lasted for 10 min at 37 °C and was stopped by adding 30 μL of sodium dodecyl sulfate solution. The absorbance (ODs) was measured at 405 nm. The absorbance (OD_0_) of reaction mixture under the same conditions without the addition of essential oil solution was also measured. The inhibition percentage (%) was calculated by the formula:

$$\mathrm{Inhibition}\;\mathrm{percentage}\;\left(\%\right)=\frac{OD_{0}-OD_{S}}{OD_{0}}\times 100\%$$

Tacrine and tetraisopropyl pyrophosphoramide (iso-OMPA) were employed as the reference for AChE and BuChE inhibitor, respectively. Essential oils at two final concentrations (20 and 2 μg/mL) were tested for a preliminary screening. The IC_50_ values (concentration of essential oils that inhibits the hydrolysis of substrates by 50%) were determined from dose–effect curves by linear regression.

### 4.8. Statistical Analysis

Extraction, GC analysis, antioxidant, antimicrobial, and cholinesterase inhibition activities assays of essential oils were performed in triplicates, and the data were expressed as the mean ± SD. SPSS 25.0 software was used for statistical analysis. Differences among samples were assessed by one-way ANOVA analysis, and a value of *p* < 0.05 was considered as indicative of statistical significance.

## 5. Conclusions

In the present study, we investigated the chemical composition, antioxidant, antimicrobial, and cholinesterase inhibitory activities of the essential oils from the leaves, stems, and roots of *K. coccinea*. Differences in the chemical composition of the essential oils from different parts of this plant have been observed. The major compounds of three essential oils were α-pinene, β-pinene, camphene, borneol, δ-cadinene, β-elemene, etc. By using three antioxidant activity evaluation methods with different mechanisms, the essential oils displayed weak antioxidant effects, indicating their potential as natural antioxidants was low. For different parts of this plant, the root oil was the most effective in antioxidant action. To our knowledge, our study is the first to report the antimicrobial and cholinesterase inhibition activities of *K. coccinea* essential oils. No obvious cholinesterase inhibition activities were displayed by the essential oils. However, the essential oils of *K. coccinea* exhibited more inhibitory effects against *S. aureus* than those of other microbial strains, indicating their potential use as a natural antibacterial agent against *S. aureus* with possible application in food and pharmaceutical industries.

## Figures and Tables

**Table 1:** Chemical compositions of the essential oils from the leaves, stems, and roots of *K. coccinea*.

No.	Compound	RI Calc.	RI Lit.	Identification	Relative Percentage (%)		
					Leaf Oil	Stem Oil	Root Oil
1	tricyclene	1011	1012	RI; MS	0.08 ± 0.01	0.27 ± 0.005	0.63 ± 0.02
2	α-pinene	1026	1025	RI; MS	42.02 ± 0.10	41.79 ± 0.83	16.60 ± 0.34
3	α-thujene	1028	1027	RI; MS	nd	nd	2.07 ± 0.13
4	α-fenchene	1058	1061	RI; MS	0.07 ± 0.00	0.09 ± 0.001	0.20 ± 0.01
5	camphene	1068	1068	RI; MS	1.56 ± 0.02	4.43 ± 0.13	10.95 ± 0.15
6	β-pinene	1113	1110	RI; MS	18.82 ± 0.08	18.71 ± 0.41	10.03 ± 0.20
7	sabinene	1123	1122	RI; MS	0.80 ± 0.003	1.10 ± 0.03	1.01 ± 0.02
8	3-carene	1150	1147	RI; MS	nd	3.39 ± 0.08	2.43 ± 0.05
9	myrcene	1164	1161	RI; MS	2.93 ± 0.02	2.89 ± 0.07	1.87 ± 0.03
10	α-phellandrene	1166	1168	RI; MS	0.20 ± 0.01	0.26 ± 0.01	0.12 ± 0.01
11	α-terpinene	1182	1178	RI; MS	0.60 ± 0.01	0.30 ± 0.01	0.19 ± 0.01
12	d-limonene	1202	1198	RI; MS	1.69 ± 0.01	1.31 ± 0.04	1.43 ± 0.02
13	β-phellandrene	1211	1209	RI; MS	2.05 ± 0.02	1.99 ± 0.05	nd
14	eucalyptol	1213	1211	RI; MS	nd	nd	2.23 ± 0.03
15	2-pentyl furan	1234	1235	RI; MS	nd	0.11 ± 0.01	nd
16	γ-terpinene	1247	1245	RI; MS	1.01 ± 0.01	0.54 ± 0.04	0.33 ± 0.01
17	(*E*)-β-ocimene	1253	1250	RI; MS	0.27 ± 0.01	0.08 ± 0.03	nd
18	*p*-cymene	1273	1270	RI; MS	0.08 ± 0.01	0.38 ± 0.005	0.38 ± 0.01
19	terpinolene	1285	1282	RI; MS	0.66 ± 0.01	0.27 ± 0.01	0.14 ± 0.00
20	α-cubebene	1459	1460	RI; MS	nd	0.15 ± 0.01	0.34 ± 0.01
21	cyclosativene	1482	1483	RI; MS	nd	0.11 ± 0.001	0.13 ± 0.01
22	α-copaene	1493	1491	RI; MS	0.89 ± 0.01	2.29 ± 0.04	3.03 ± 0.04
23	β-cubebene	1540	1542	RI; MS	nd	0.06 ± 0.00	0.18 ± 0.01
24	linalool	1547	1543	RI; MS	0.07 ± 0.001	nd	nd
25	isoledene	1553	1559	RI; MS	nd	nd	0.13 ± 0.01
26	bornyl acetate	1583	1579	RI; MS	0.14 ± 0.002	0.46 ± 0.02	0.60 ± 0.05
27	fenchol	1584	1575	RI; MS	0.39 ± 0.01	nd	nd
28	β-elemene	1593	1591	RI; MS	4.45 ± 0.05	2.04 ± 0.07	1.86 ± 0.04
29	β-caryophyllene	1600	1598	RI; MS	2.87 ± 0.04	2.71 ± 0.10	3.08 ± 0.09
30	terpinen-4-ol	1605	1601	RI; MS	1.69 ± 0.03	1.45 ± 0.11	1.49 ± 0.06
31	α-humulene	1673	1667	RI; MS	0.90 ± 0.02	0.86 ± 0.05	0.99 ± 0.03
32	γ-muurolene	1691	1690	RI; MS	0.24 ± 0.004	0.16 ± 0.01	0.32 ± 0.01
33	γ-curcumene	1693	1692	RI; MS	0.08 ± 0.01	nd	nd
34	α-terpineol	1700	1694	RI; MS	1.67 ± 0.04	0.64 ± 0.06	0.36 ± 0.01
35	borneol	1704	1700	RI; MS	0.50 ± 0.01	0.63 ± 0.06	7.71 ± 0.24
36	germacrene D	1713	1708	RI; MS	nd	0.28 ± 0.02	nd
37	bicyclosesquiphellandrene	1715	1706	RI; MS	0.12 ± 0.01	0.39 ± 0.03	0.99 ± 0.06
38	β-selinene	1724	1717	RI; MS	1.62 ± 0.05	1.29 ± 0.09	1.90 ± 0.12
39	α-selinene	1729	1725	RI; MS	1.75 ± 0.05	0.87 ± 0.08	1.07 ± 0.04
40	β-curcumene	1744	1737	RI; MS	0.26 ± 0.004	nd	0.50 ± 0.03
41	δ-cadinene	1761	1756	RI; MS	1.52 ± 0.05	3.06 ± 0.03	7.06 ± 0.38
42	γ-cadinene	1764	1763	RI; MS	0.52 ± 0.02	nd	nd
43	citronellol	1767	1764	RI; MS	0.09 ± 0.005	nd	nd
44	cis-cadina-1(2),4-diene	1784	1788	RI; MS	0.13 ± 0.005	nd	0.45 ± 0.03
45	germacrene B	1830	1824	RI; MS	0.13 ± 0.01	nd	nd
46	cis-calamenene	1839	1834	RI; MS	0.01 ± 0.00	0.07 ± 0.01	0.15 ± 0.01
47	epicubebol	1893	1900	RI; MS	nd	0.14 ± 0.03	1.00 ± 0.06
48	α-calacorene	1925	1921	RI; MS	nd	nd	0.11 ± 0.01
49	cubebol	1944	1942	RI; MS	nd	0.17 ± 0.02	1.70 ± 0.12
50	caryophyllene oxide	1991	1986	RI; MS	nd	0.12 ± 0.03	0.38 ± 0.12
51	(*E*)-nerolidol	2042	2036	RI; MS	1.52 ± 0.03	0.36 ± 0.06	1.38 ± 0.11
52	humulene epoxide II	2051	2047	RI; MS	nd	nd	0.22 ± 0.00
53	cubenol	2066	2068	RI; MS	0.17 ± 0.005	0.28 ± 0.04	1.10 ± 0.06
54	1,10-di-epi-cubenol	2073	2074	RI; MS	0.23 ± 0.01	0.45 ± 0.09	1.71 ± 0.11
55	globulol	2082	2082	RI; MS	0.09 ± 0.00	nd	nd
56	guaiol	2094	2089	RI; MS	0.15 ± 0.004	nd	nd
57	spathulenol	2131	2127	RI; MS	0.09 ± 0.01	0.08 ± 0.02	1.05 ± 0.06
58	τ-cadinol	2177	2175	RI; MS	0.21 ± 0.01	0.12 ± 0.02	0.40 ± 0.02
59	trans-muurolol	2205	2209	RI; MS	0.21 ± 0.01	0.19 ± 0.04	0.73 ± 0.05
60	farnesol isomer	2356	2357	RI; MS	0.11 ± 0.02	nd	nd
							
	Compounds identified				46	44	47
	Total identified (%)				95.66	97.35	92.72
	Monoterpene hydrocarbons				72.84	77.8	48.38
	Oxygenated monoterpenes				4.41	2.72	11.79
	Sesquiterpene hydrocarbons				15.49	14.34	22.29
	Oxygenated sesquiterpenes				2.78	1.91	9.67
	Others				0.14	0.57	0.6

Note: RI Calc.: Retention indices calculated against *n*-alkane series on InertCap WAX column; RI Lit.: Retention indices from literature on similar columns with the same polarity; MS: Mass Spectrum; nd: not detected.

**Table 2:** Antioxidant activity of *K. coccinea* essential oils.

	DPPH	ABTS	FRAP
	μmol TE /g	μmol TE /g	μmol TE /g
Leaf Oil	0.92 ± 0.06	7.97 ± 0.41	30.89 ± 1.11
Stem Oil	1.52 ± 0.11	15.26 ± 0.72	34.77 ± 1.46
Root Oil	4.21 ± 0.05	25.52 ± 0.22	48.79 ± 1.08
BHT	2071.96 ± 15.54	5028.80 ± 41.66	2492.60 ± 25.33

NA: not active.

**Table 3:** Antimicrobial activity of essential oils of *K. coccinea*, given as MIC (mg/mL) and MMC (mg/mL).

Species	Leaf Oil		Stem Oil		Root Oil		Gentamycin Sulfate		Amphotericin B	
	MIC	MMC	MIC	MMC	MIC	MMC	MIC	MMC	MIC	MMC
*S. aureus*	1.56	3.13	1.56	3.13	0.78	1.56	0.016	0.016	/	/
*S. lentus*	3.13	6.25	3.13	6.25	3.13	6.25	0.004	0.004	/	/
*B. atrophaeus*	50.0	100.0	100.0	100.0	25.0	50.0	0.0003	0.0003	/	/
*p. aeruginosa*	6.25	12.5	6.25	12.5	6.25	12.5	0.002	0.002	/	/
*E. coli*	6.25	12.5	6.25	12.5	6.25	12.5	0.016	0.016	/	/
*C. albicans*	3.13	6.25	3.13	6.25	3.13	6.25	/	/	0.0004	0.0008

**Table 4:** Cholinesterase inhibition activity of *K. coccinea* essential oils.

	Concentratio (μg/mL)	Acetylcholinesterase Inhibition (%)	Butyrylcholinesterase Inhibition (%)
Leaf Oil	20	4.80 ± 0.23	NA
	2	NA	NA
Stem Oil	20	5.44 ± 0.11	1.68 ± 0.14
	2	NA	NA
Root Oil	20	0.83 ± 0.10	NA
	2	NA	NA
Tacrine	0.33	55.39 ± 2.79	/
Iso-OMPA	0.86	/	48.54 ± 6.00

## Data Availability

The data presented in this study are available on request from the corresponding author. The data are not publicly available at this time as it also forms part of an ongoing study.

## Sample Availability

Samples of the leaves, stems and roots of *K. coccinea* are available from the authors.
